# Basolateral Amygdala to Nucleus Accumbens Communication Differentially Mediates Devaluation Sensitivity of Sign- and Goal-Tracking Rats

**DOI:** 10.3389/fnbeh.2020.593645

**Published:** 2020-11-25

**Authors:** Daniel E. Kochli, Sara E. Keefer, Utsav Gyawali, Donna J. Calu

**Affiliations:** ^1^Department of Anatomy and Neurobiology, University of Maryland School of Medicine, Baltimore, MD, United States; ^2^Program in Neuroscience, University of Maryland School of Medicine, Baltimore, MD, United States

**Keywords:** basolateral amygdala, nucleus accumbens, flexibility, sign-tracking, goal-tracking, devaluation

## Abstract

Rats rely on communication between the basolateral amygdala (BLA) and nucleus accumbens (NAc) to express lever directed approach in a Pavlovian lever autoshaping (PLA) task that distinguishes sign- and goal-tracking rats. During PLA, sign-tracking rats preferentially approach an insertable lever cue, while goal-tracking rats approach a foodcup where rewards are delivered. While sign-tracking rats inflexibly respond to cues even after the associated reward is devalued, goal-tracking rats flexibly reduce responding to cues during outcome devaluation. Here, we sought to determine whether BLA–NAc communication, which is necessary for sign, but not goal-tracking, drives a rigid appetitive approach of sign-tracking rats that are insensitive to manipulations of outcome value. Using a contralateral chemogenetic inactivation design, we injected contralateral BLA and NAc core with inhibitory DREADD (hm4Di-mCherry) or control (mCherry) constructs. To determine sign- and goal-tracking groups, we trained rats in five PLA sessions in which brief lever insertion predicts food pellet delivery. We sated rats on training pellets (devalued condition) or chow (valued condition) before systemic clozapine injections (0.1 mg/kg) to inactivate BLA and contralateral NAc during two outcome devaluation probe tests, in which we measured lever and foodcup approach. Contralateral BLA–NAc chemogenetic inactivation promoted a flexible lever approach in sign-tracking rats but disrupted the flexible foodcup approach in goal-tracking rats. Consistent with a prior BLA–NAc disconnection lesion study, we find contralateral chemogenetic inactivation of BLA and NAc core reduces lever, but not the foodcup approach in PLA. Together these findings suggest rigid appetitive associative encoding in BLA–NAc of sign-tracking rats hinders the expression of flexible behavior when outcome value changes.

## Introduction

A body of evidence suggests that sign- and goal-tracking differences predict vulnerability to Substance Use Disorder (SUD; Tomie et al., 2008; Flagel et al., 2009; Saunders and Robinson, 2010; Saunders et al., 2013; Yager et al., 2015; Kawa et al., 2016; Villaruel and Chaudhri, 2016). Reward predictive cues acquire appetitive motivational properties; a psychological process often referred to as incentive salience that is postulated to drive SUD vulnerability (Berridge, 1996; Robinson and Berridge, 1993; Berridge and Robinson, 2016). Sign-tracking (ST) and goal-tracking (GT) individual differences during a Pavlovian lever autoshaping (PLA) task capture the degree to which reward predictive cues acquire incentive salience (Flagel et al., 2009; Pitchers et al., 2015; Flagel and Robinson, 2017) and predict heightened drug-cue induced relapse despite negative consequences (Saunders and Robinson, 2010; Saunders et al., 2013). Before drug experience, ST rats inflexibly respond to cues after reward devaluation (Morrison et al., 2015; Nasser et al., 2015; Patitucci et al., 2016; Smedley and Smith, 2018; Keefer et al., 2020). A prior lesion study indicates that communication between the basolateral amygdala (BLA) and nucleus accumbens (NAc) is necessary for the acquisition and expression of a lever approach that classifies ST rats (Chang et al., 2012). Here, we aim to determine the extent to which the incentive salience process supported by BLA–NAc core communication interferes with the expression of devaluation sensitivity in ST rats.

Given tracking-related behavioral differences in incentive salience processing and devaluation sensitivity, we hypothesize that the BLA to NAc communication drives a rigid lever cue approach in ST rats and outcome value-sensitive foodcup behavior in GT rats. BLA and NAc are critically involved in Pavlovian incentive learning processes including second-order conditioning (SOC) and outcome devaluation. SOC is a learning process that relies upon the positive incentive value of the conditioned stimulus (CS), while outcome devaluation relies upon the current value of the unconditioned stimulus (US; Holland and Rescorla, 1975). Pre-training lesions of either BLA or NAc impair both SOC and outcome devaluation, while post-training lesions of BLA disrupt only outcome devaluation, but not SOC (Hatfield et al., 1996; Setlow et al., 2002; Johnson et al., 2009; Singh et al., 2010). Instead, the expression of SOC is mediated by NAc (McDannald et al., 2013). Pre-training, contralateral lesions disconnecting the BLA and NAc impair both SOC (Setlow et al., 2002) and lever approach (the approach response characterizing ST rats), while leaving intact food cup-directed behavior (the approach response characterizing GT rats; Chang et al., 2012). Taken together, the BLA and NAc support incentive learning relying on both conditioned stimulus (CS) value and current outcome (US) value. A growing number of studies demonstrate that GT, but not ST, rats flexibly reduce approach after outcome devaluation induced by satiety or illness (Morrison et al., 2015; Nasser et al., 2015; Patitucci et al., 2016; Smedley and Smith, 2018; Rode et al., 2020; Keefer et al., 2020). Both ST and GT rats similarly acquire and express SOC (Nasser et al., 2015; Saddoris et al., 2016), learning that requires BLA–NAc communication. SOC can be acquired *via* two “strategies;” either *via* mapping incentive value onto the cue itself, or by the cue invoking a representation of the outcome value. We posit that sign- and goal-trackers may utilize underlying BLA–NAc circuitry to differentially mediate incentive learning relying on CS or US value, respectively. Because devaluation specifically affects the value of the outcome, it allows us to parse out this distinction.

The primary prediction of our hypothesis is that contralateral chemogenetic inactivation of BLA and NAc core will make ST rats more flexible in outcome devaluation. Specifically, in intact ST rats, we expect similar levels of responding for valued and devalued conditions, consistent with our prior reports (Nasser et al., 2015; Keefer et al., 2020). However, with BLA–NAc inactivation we predict a reduced lever-directed approach for devalued relative to valued conditions. We expressed inhibitory DREADDs in contralateral BLA and NAc core and use systemic injections of low-dose clozapine to inactivate these structures during outcome-specific satiety devaluation. Because of the unidirectional and predominately unilateral projections of BLA to NAc (Swanson and Cowan, 1975; Ottersen, 1980; Russchen and Price, 1984; Heimer et al., 1991; Brog et al., 1993; Kelley et al., 1993), contralateral inactivation of these structures disrupts communication from BLA to NAc core, while leaving an intact BLA and NAc core to support behavior that relies on either of these structures alone.

## Materials and Methods

### Subjects and Apparatus

We maintained male and female Long-Evans rats (Charles River Laboratories, Wilmington, MA, USA; 250–275 g at the time of arrival; *N* = 107) on a reverse 12 h light/dark cycle (lights off at 9:00 AM). We conducted all behavioral training and testing during the dark phase of the cycle. All rats had *ad libitum* access to water and standard laboratory chow before being individually housed after surgical procedures. After recovery, we food-restricted rats and maintained them at ~90% of their baseline body weight throughout the experiment. We performed all experiments following the “Guide for the Care and Use of Laboratory Animals” (8th edition, 2011, US National Research Council) and were approved by the University of Maryland, School of Medicine Institutional Animal Care and Use Committee (IACUC).

Prior to any training, we performed intracranial viral injection surgeries to deliver AAV8.hSyn.hM4Di.mCherry (hM4Di) or AAV8.hSyn.mCherry (mCherry) targeting the BLA and contralateral NAc core. We excluded 37 rats from subsequent analyses due to poor health, premature death, or poor/misplaced viral expression based on histological analysis (Figure 4). Twenty-eight intermediate rats were excluded because they did not express a preferred conditioned response (lever or foodcup contact) to classify as sign- or goal-tracking rats, resulting in 42 rats being included in our analyses. The PCA characterization completed after surgery for viral injections resulted in the following number of rats in each group: ST *n* = 20, mCherry *n* = 9 (*n* = 5 females, *n* = 4 males), hM4Di *n* = 11 (*n* = 7 females, *n* = 4 males), and GT *n* = 22, mCherry *n* = 10 (*n* = 4 females, *n* = 6 males), hM4Di *n* = 12 (*n* = 7 females, *n* = 5 males).

**Figure 1:**
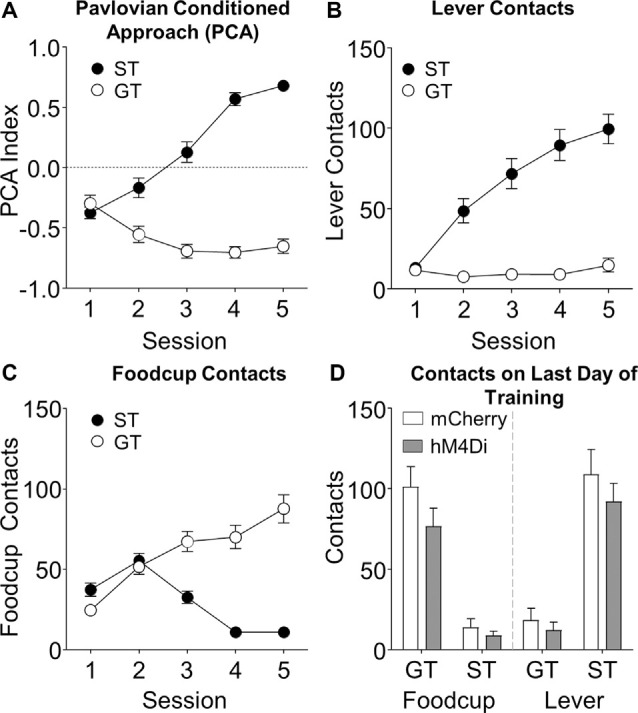
Pavlovian lever autoshaping (PLA) acquisition data. Data represents **(A)** average Pavlovian Conditioned Approach (PCA) score, **(B)** lever contacts, **(C)** foodcup contacts during training; and **(D)** both lever and foodcup contacts on fifth training session are represented as a function of viral condition; there were no differences as a function of virus (*p*s > 0.05).

**Figure 2:**
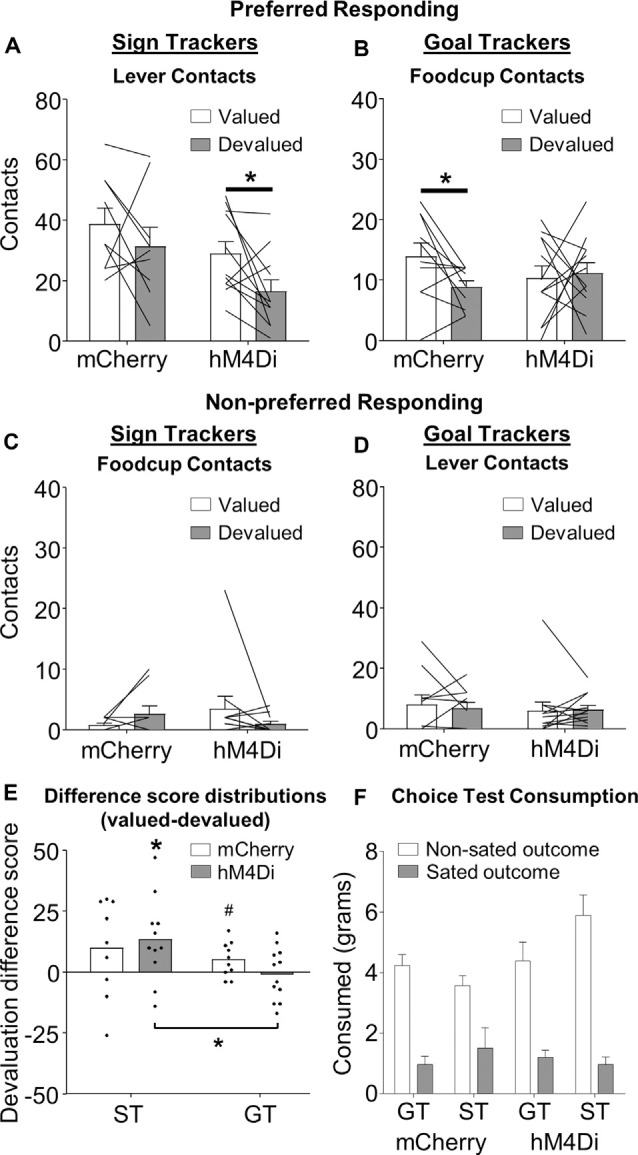
Outcome devaluation in sign- and goal-tracking rats. Data represent individual subjects (line) and group averaged (bars) for **(A,B)** preferred responding (ST: lever contact, GT: foodcup contact) and **(C,D)** non-preferred responding (ST: foodcup contact, GT: lever contact), + SEM. *A priori* planned comparisons reveal that **(A)** hM4Di (*t*_(10)_ = 2.582, *p < 0.05), but not mCherry (*t*_(8)_ = 1.495, *p* = 0.173), ST rats show devaluation effect (difference between valued and devalued) for lever directed behavior. **(B)** mCherry (*t*_(9)_ = 2.273 *p < 0.05), but not hM4Di (*t*_(11)_ = 0.270, *p* = 0.792), GT rats show devaluation effect for foodcup directed behavior. No interactions were observed for non-preferred responding. **(E)** Population distributions of devaluation difference scores (valued preferred responding–devalued preferred responding). ST hM4Di population distribution was significantly shifted above zero and significantly different than the GT hM4Di population distribution (*p < 0.05, ^#^*p* = 0.07). **(F)** Consumption during the choice test following outcome devaluation is represented as a function of tracking and viral condition.

**Figure 3:**
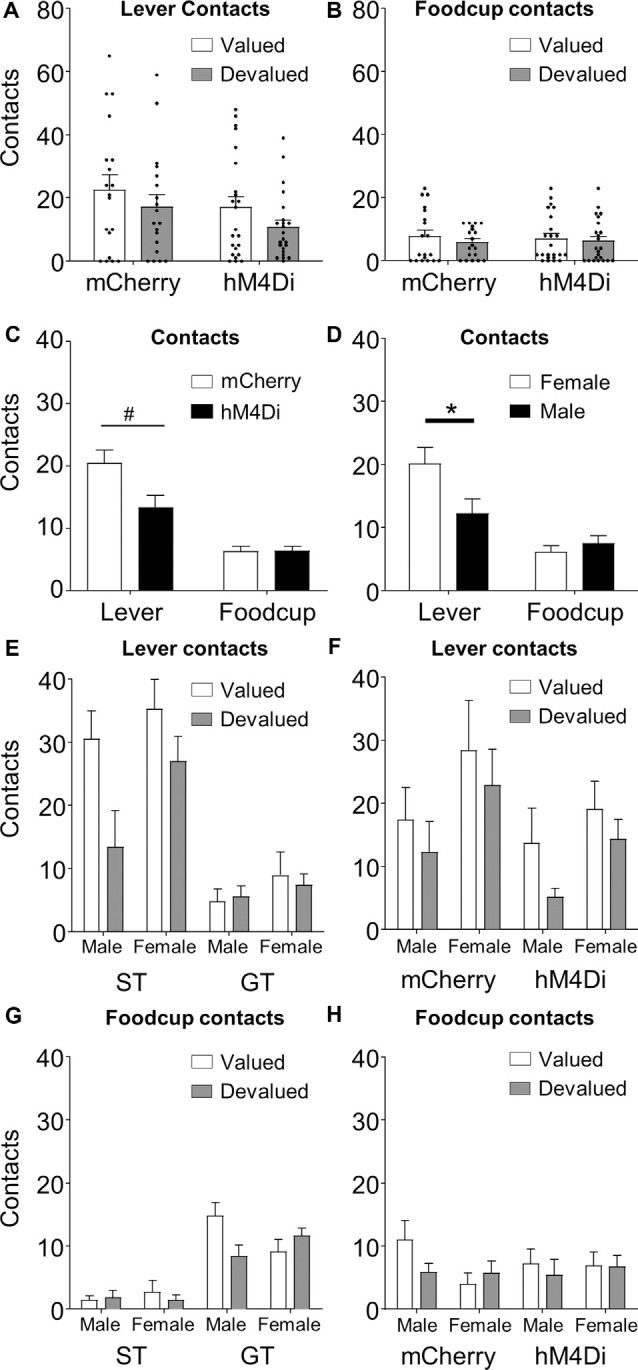
Lever and foodcup contact data represent individual subjects (dot) and group averages (bars) + SEM for **(A)** lever **(B)** and foodcup contacts during outcome devaluation, collapsed across Tracking Group. **(C)** Data represent contacts collapsed across Tracking Group and Value; basolateral amygdala (BLA)–nucleus accumbens (NAc) core inactivation disrupts lever but not foodcup approach, *F*_(1,34)_ = 4.484, *p* = 0.042. *Post hoc* tests revealed a trend toward an effect of inactivation on lever-directed responding, such that hM4Di rats pressed less than mCherry rats, *t*_(22)_ = 2.05, ^#^*p* = 0.053. Sex effects split by Response type **(D)**, Tracking Group **(E,G)**, and Virus group **(F,H)**. Data represent group averages (bars) + SEM. **(D)** Females perform more lever-directed responses than males during outcome devaluation tests overall. **(G)** Tracking Group × Value × Sex interaction of foodcup responding (*F*_(1,34)_ = 5.02, *p* = 0.032). *Indiciates significant difference with *p* < 0.05.

**Figure 4:**
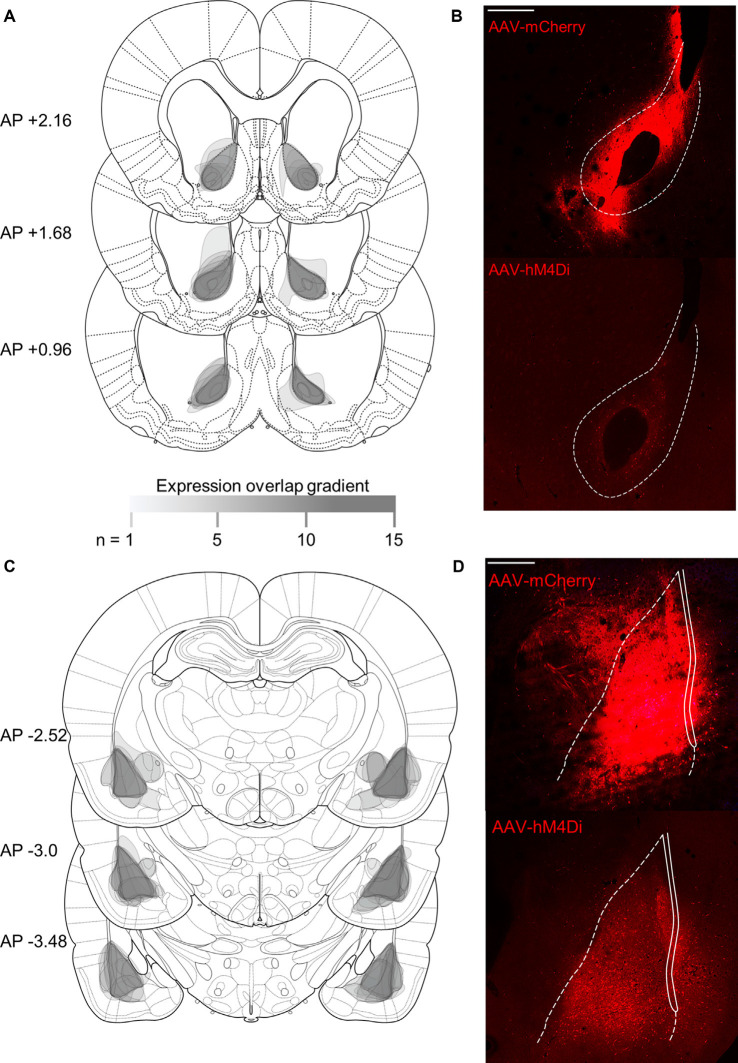
Histological verification of viral expression in NAc core and BLA. Rats were injected with viral constructs unilaterally in BLA and in contralateral NAc core (mm from Bregma; Paxinos and Watson, 2007); scale bars represent 500 μm. Unilateral expression was counterbalanced, but the expression is shown in both hemispheres. **(A)** Schematic representation of viral expression and **(B)** representative image of mCherry (top) and hM4Di (bottom) NAc core expression. **(C)** Schematic representation of viral expression and **(D)** representative image of (top) mCherry and hM4Di (bottom) BLA expression. The legend indicates the density of overlapping expression, where (*n*) is the number of overlapping cases to produce the represented opacity.

We conducted behavioral experiments in individual standard experimental chambers (25 × 27 × 30 cm; Med Associates) located outside of the colony room. Each chamber was housed in an individual sound-attenuating cubicle with a ventilation fan. During PLA and devaluation probe tests, each chamber had one red house light (6 W) located at the top of the wall that was illuminated for the duration of each session. The opposite wall of the chamber had a recessed foodcup (with photo beam detectors) located 2 cm above the grid floor. The foodcup had an attached programmed pellet dispenser to deliver 45 mg food pellets (catalog# 1811155; Test Diet Purified Rodent Tablet (5TUL); protein 20.6%, fat 12.7%, carbohydrate 66.7%). These pellets are designed to be highly-palatable and are not only discriminable from ordinary chow but are preferred to most other reinforcers, including 91% sucrose pellets (Pickens et al., 2012). One retractable lever was positioned on either side of the foodcup, counterbalanced between subjects, 6 cm above the floor. Sessions began with the illumination of the red house light and lasted ~26 min.

### Surgical Procedures

We rapidly anesthetized rats with 5% isoflurane and maintained them at 2–3% isoflurane (Vetone, Boise, ID, USA) throughout the procedure. We maintained body temperature with a heating pad during the procedure. Before the first incision, we administered a subcutaneous injection of the analgesic carprofen (5 mg/kg) and subdermal injection of the local anesthetic lidocaine (10 mg/ml at the incision site). We secured rats in the stereotaxic apparatus (model 900, David Kopf Instruments, Tujunga, CA, USA) and leveled the skull by equating lambda and Bregma in the dorsal-ventral plane. We lowered 10 μl Hamilton syringes (Hamilton, Reno, NV, USA) into the brain targeting the BLA and contralateral NAc core (counterbalanced) using the following coordinates: BLA: (AP −3.0 mm, ML ± 5.0 mm, and DV −8.6 mm 0° from midline) NAc core: (AP +1.8 mm, ML ± 2.5 mm, and DV −7.0 mm −6° from midline) relative to Bregma skull surface (Paxinos and Watson, 2007). We delivered AAV8.hSyn.hM4Di.mCherry (hM4Di) or AAV8.hSyn.mCherry (mCherry) targeting the BLA and contralateral NAc core (Addgene, Watertown, MA, USA) *via* a micropump (UltraMicroPump III, World Precision Instruments, Sarasota, FL, USA) at a volume of 600 nl per site at a rate of 250 nl/min. We left syringes in place for 10 min after the infusion ended to allow diffusion of the viral constructs before suturing incisions. After surgery, we placed the rats into a recovery cage on a heating pad until ambulatory. We administered Carprofen (5 mg/kg; s.c.) 24 and 48 h post-surgery and monitored weights daily to confirm recovery.

### Pavlovian Lever Autoshaping Training and Testing

We trained rats over five daily PLA sessions (approximately 26 min duration per session), which consisted of 25 reinforced lever conditioned stimulus (CS+) presentations occurring on a variable time (VT) 60 s schedule (50–70 s). Trials consisted of the insertion of a retractable lever (left or right, counterbalanced) for 10 s, after which the lever was retracted and two 45 mg food pellets were delivered to the foodcup, non-contingent on rat behavior. The sessions took place in darkness with a red house light that was illuminated for the duration of the session.

After the acquisition, we performed 2 days of satiety-induced outcome devaluation testing. Before test sessions, we gave rats free homecage access to 30 g of rat chow (valued condition) or the same food pellets delivered during training (devalued condition) in a pre-habituated ceramic ramekin (similar to Parkes and Balleine, 2013). Immediately following satiation, we gave systemic injections of 0.1 mg/kg clozapine i.p. (Tocris, Bristol, UK) dissolved in bacteriostatic saline before transport to the behavioral chambers (Gomez et al., 2017). We waited 30 min after injection to allow binding of the ligand to the DREADD receptors. Then we gave a PLA probe test (approximately 10 min duration) consisting of 10 non-reinforced lever presentations occurring on a VT60 s schedule (50–70 s). Immediately following the testing, we gave rats a 30 min choice test in which they could consume up to 10 g each of rat chow or pellets in the homecage. Between each PLA test, we gave rats a single reinforced lever autoshaping training session to track stability in Pavlovian behavior. The next day, we gave rats a second round of satiety devaluation, PLA probe, and choice tests while sated under the opposite condition (pellet or chow; order counterbalanced).

### Measurements

During the PLA acquisition and probe tests, we collected three behavioral measurements during the 10 s CS (lever) period. All behavioral measurements were automatically collected and scored *via* MED-PC computer software (Med Associates, Georgia, VT, USA). While lever and foodcup interactions have unique motor features, including lever deflection (press, bite and lick) and foodcup photobeam break (head entry, bite and lick), we describe both lever and foodcup measures as “contact” to capture a common feature of both approach responses. For a lever response or foodcup response to be recorded by the automated system, contact must occur. For foodcup and lever contacts, we recorded the total number of contacts and latency to the first contact for all sessions. On trials in which no contact occurred, we recorded a latency value of 10 s. We calculated the lever or foodcup probabilities by dividing the number of trials that a lever or foodcup contact was made by the total number of trials in the session.

The criterion used for behavioral characterization of sign- and goal- tracking phenotype was based on a Pavlovian Conditioned Approach (PCA) analysis (Meyer et al., 2012) determined by averaging PCA scores during training sessions four and five. The PCA score quantifies the variation between lever directed (sign-tracking) and foodcup directed (goal-tracking) behaviors. Each rat’s PCA score is the average of three difference score measures (each ranging from −1.0 to +1.0): (1) preference score, (2) latency score, and (3) probability score. The preference score is the number of lever presses during the CS, minus the foodcup pokes during the CS, divided by the sum of these two measures. The latency score is the average latency to make a foodcup poke during the CS, minus the latency to lever press during the CS, divided by the duration of the CS (10 s). The probability score is the probability to lever press, minus the probability to foodcup poke observed throughout the session. Generally speaking, sign-tracking rats show a preferred conditioned response of lever contact, which is performed at a shorter latency and higher probability than foodcup contacts during the lever cue presentation. Goal-tracking rats show a preferred conditioned response of foodcup contact, which is performed at a shorter latency and higher probability than foodcup contacts during the lever cue presentation. Intermediate rats do not show a preferred conditioned response, performing similar amounts of lever and foodcup contact, performed at similar latencies and probabilities during lever cue presentation. Sign-tracking PCA scores range from +0.33 to +1.0, goal-tracking PCA scores range from −0.33 to −1.0, and intermediate group PCA scores range from −0.32 to +0.32 (intermediates were not included in reported analyses).

### Histology

After completion of behavioral testing, we deeply anesthetized rats with isoflurane and transcardially perfused them with 100 ml of 0.1 M PBS followed by 400 ml 4% paraformaldehyde in 0.1 M sodium phosphate, pH 7.4. We removed brains and post-fixed them in 4% paraformaldehyde for 2 h before transfer to a 30% sucrose 4% paraformaldehyde solution in 0.1 M sodium phosphate for 48 h at 4°C. We then rapidly froze them *via* dry ice and stored them at −20°C until sectioning. We collected 50 μm coronal sections through the entire extent of the NAc and amygdala *via* a cryostat (Lecia Microsystems). We mounted sections on slides and verified viral expression in BLA and NAc core using anatomical boundaries defined by Paxinos and Watson (Paxinos and Watson, 2007) using a confocal microscope. The observer was blind to the condition and behavior of each animal.

### Experimental Design and Statistical Analysis

Data were analyzed using SPSS statistical software (IBM v.25) with mixed-design repeated-measures ANOVAs. Analyses included the within-subjects factors of Response (foodcup, lever) and Value (valued, devalued) and the between-subject factors of Virus (mCherry, hM4Di), Tracking Group (ST, GT), and Sex (female, male) as indicated in the results section. Unplanned *post hoc* tests used a Bonferroni correction. All analyses include only ST and GT rats to test *a priori* hypotheses based on previously reported devaluation sensitivity differences in these two tracking groups (Keefer et al., 2020; Nasser et al., 2015). We also calculated devaluation difference scores (valued preferred responding-devalued preferred responding) for each viral/tracking group and performed Wilcoxon signed-rank and rank-sum statistics on these distributions. We report population means (μ) and significance values for distribution shifts from zero (signed rank) or comparing distributions (rank-sum). We include both males and females in our study and report effect sizes in our analyses in which Sex is included as a factor (Miller et al., 2017). Effect sizes are expressed as partial η^2^. This measure of effect size is calculated as $\eta_{\text{p}}^{2}$ = SSeffect/(SSeffect + SSerror), where SSeffect is the sum of squares of the effect and SSerror is the sum of squares of the error term.

## Results

### Acquisition of Pavlovian Lever Autoshaping

We trained rats for 5 days in PLA to determine tracking groups before outcome devaluation testing. We used a Pavlovian Conditioned Approach Index (Figure 1A, see methods for calculation) that takes into account the number of lever and foodcup contacts (Figures 1B,C), latency to contact, and the probability of contact for both lever and foodcup. We analyzed the lever autoshaping training data using six separate mixed-design, repeated measures ANOVAs with the between-subjects factor of Tracking Group (ST, GT) and the within-subjects factors of Session (1–5). In Table 1, we report the main effects and interactions of these analyses. Notably, the critical Session × Tracking Group interactions were significant for all six measures of conditioned responding (*F*s > 10.836, *p*s < 0.001). We analyzed levels of lever and foodcup contacts on the last day of training, using between-subject factors of Virus (mCherry, hM4Di) and Tracking Group (ST, GT) and found no Virus main effects nor Virus × Tracking Group interactions (Figure 1D) indicating that behavior did not differ between viral conditions before the test for any of the six lever autoshaping measures (*F*s < 2.48, *p*s > 0.05).

**Table 1:** Repeated measures analysis of variance (ANOVA) for Pavlovian lever autoshaping across all tracking groups.

Effect	Degrees of freedom	Contact		Latency		Probability	
		*F*	*p*	*F*	*p*	*F*	*p*
**Lever**
Session	(4,160)	27.945	<0.001	33.552	<0.001	29.912	<0.001
Tracking Group	(1,40)	85.065	<0.001	76.816	<0.001	105.851	<0.001
Session * Tracking Group	(4,160)	25.944	<0.001	20.843	<0.001	20.738	<0.001
**foodcup**
Session	(4,160)	10.836	<0.001	26.314	<0.001	23.370	<0.001
Tracking Group	(1,40)	28.905	<0.001	54.610	<0.001	40.029	<0.001
Session * Tracking Group	(4,160)	49.324	<0.001	77.611	<0.001	55.551	<0.001

### Effects of Contralateral BLA–NAc Core Inactivation on Pavlovian Approach During Outcome Devaluation

We hypothesized that ST rats rely on BLA–NAc core to drive rigid appetitive approach. To test this* a priori* hypothesis, we examined the extent to which BLA–NAc core contralateral chemogenetic inactivation altered the preferred response of ST rats during satiety devaluation tests. For ST rats the preferred response is lever contacts (Figure 2A), while for GT rats the preferred response is foodcup contacts (Figure 2B). Notably, mCherry ST control rats showed no difference in lever contact between valued and devalued tests (*t*_(8)_ = 1.495, *p* = 0.173), confirming their insensitivity to devaluation, consistent with prior reports (Keefer et al., 2020; Nasser et al., 2015). ST rats expressing hM4Di showed greater lever contact during valued compared to devalued tests (*t*_(10)_ = 2.582, *p* = 0.027), indicating devaluation sensitivity in ST rats with contralateral chemogenetic inactivation of BLA–NAc core (Figure 2A). In contrast, mCherry GT control rats showed greater foodcup contact during valued compared to devalued tests (*t*_(9)_ = 2.273 *p* = 0.049), confirming their devaluation sensitivity that is consistent with prior reports (Keefer et al., 2020; Nasser et al., 2015). GT rats expressing hM4Di constructs showed no difference in foodcup contact during valued compared to devalued tests (*t*_(11)_ = 0.270, *p* = 0.792), indicating contralateral chemogenetic inactivation of BLA–NAc core makes GT rats insensitive to devaluation (Figure 2B). We also conducted a repeated-measures ANOVA on these preferred response data using between-subjects factors of Virus (mCherry, hM4Di) and Tracking Group (GT, ST), and the within-subject factor of Value (valued, devalued). We observed main effects of Virus (*F*_(1,38)_ = 5.485, *p* = 0.025) and Tracking Group (*F*_(1,38)_ = 42.461, *p* < 0.001), as well as Value × Tracking Group (*F*_(1,38)_ = 4.552, *p* = 0.039) and Virus × Tracking Group (*F*_(1,38)_ = 4.460, *p* = 0.041) interactions (see Figures 2A,B), indicating both virus and value manipulations differ by tracking group. For parallel analyses of non-preferred responding (lever contact for GT and foodcup contact for ST rats), we observed a main effect of Tracking Group such that GT performed more non-preferred approach behavior, *F*_(1,38)_ = 7.773, *p* = 0.008), but no other main effects or interactions, *p*s > 0.05 (see Figures 2C,D); the lack of effects is almost certainly due to a floor effect.

We calculated difference scores (valued preferred responding-devalued preferred responding) for each viral/tracking group and performed Wilcoxon signed-rank and rank-sum statistics on the distributions of difference scores (Figure 2E). We observed overall, the distribution of devaluation scores was significantly shifted above zero (*μ* = 6.5, *p* = 0.01), consistent with the main effect of Value observed in our conventional parametric statistical tests performed on raw data. This confirms overall the devaluation task we use yields a devaluation effect (greater preferred responding during valued compared to devalued tests). We also observe the ST hM4Di distribution of difference scores was shifted significantly above zero (*μ* = 13.4; *p* = 0.02), while the ST mCherry distribution was not (*μ* = 9.9; *p* > 0.15). While the GT mCherry distribution of difference scores was trending towards significantly shifted above zero (*μ* = 5.1; *p* = 0.07), GT hM4Di distribution of difference scores was no different than zero (*μ* = −0.833, *p* = 0.79). Rank sum tests comparing distributions within the tracking group did not identify significant differences in devaluation difference scores, however, the GT hM4Di and ST hM4Di distributions were significantly different from one another (see μ-values above, *p* = 0.034), again suggesting manipulation of BLA–NAc had effects in opposite directions for ST and GT rats.

We recorded pellet and chow consumption during satiety (pre-test) and choice test (post-test). Before devaluation test sessions, we found no difference in the amount of food consumed between tracking or viral groups during the satiation hour (*F* < 1, *p* > 0.4). To confirm the devaluation of the sated food, we gave rats a post-satiety choice test following the devaluation test. Rats preferred to consume food they were not sated on, as indicated by a main effect of Outcome (sated, non-sated), *F*_(1,34)_ = 86.312, *p* < 0.0001. There were no main effects of Virus (mCherry, hM4Di), Tracking Group (ST, GT), or Test Order (chow first, pellet first; *F* < 3.9, *p* > 0.5) or interaction of these factors with Outcome (*F* < 1.4, *p* > 0.3), indicating that for both viral conditions, ST and GT have a similar preference for the non-sated food during the choice test (Figure 2F).

A prior lesion study demonstrated that BLA–NAc communication drives lever directed, but not foodcup directed behavior in lever autoshaping (Chang et al., 2012). To evaluate whether the pattern of results using our contralateral chemogenetic inactivation approach was similar to the prior BLA–NAc lesion finding, we analyzed the data by including Response (lever, foodcup) as a factor, which is shown in (Figures 3A,B). We observed a Response × Virus interaction (*F*_(1,34)_ = 4.484, *p* = 0.042), shown in Figure 3C, collapsed across tracking groups and value conditions. The interaction reveals that the lever approach is more affected by contralateral chemogenetic BLA–NAc inactivation than the foodcup approach, similar to the pattern of results observed in the BLA–NAc cross lesion study of Chang et al. (2012).

Because we included both males and females in this study, we next examined whether Sex interacted with any other factors during our devaluation tests. In addition to main effects for all factors (Value, Response, Virus, Sex, and Tracking Group, all *F* > 4.983, *p* < 0.05, all $\eta_{\text{p}}^{2}$ > 0.128), we also observed a Response × Sex interaction (Figure 3D, *F*_(1,34)_ = 4.688, *p* = 0.037, $\eta_{\text{p}}^{2}$ = 0.121), which we explore by separately analyzing each response. We analyzed lever-directed behavior with between-subjects factors of Tracking Group (ST, GT), Virus (mCherry, hM4Di), and Sex (female, male), and within-subjects factor of Value (valued, devalued). We observed main effects of Value (*F*_(1,34)_ = 8.527, *p* = 0.006, $\eta_{\text{p}}^{2}$ = 0.201) and Virus (*F*_(1,34)_ = 6.114, *p* = 0.019, $\eta_{\text{p}}^{2}$ = 0.152) previously reported. Again, we observed a main effect of Sex (*F*_(1,34)_ = 5.549, *p* = 0.024, $\eta_{\text{p}}^{2}$ = 0.140), driven primarily by more lever approach in females compared to males across the virus groups and value conditions (Figure 3D). For lever-directed behavior, there were no significant interactions with Sex, and associated effect sizes were small (Figures 3E,F; all *F* < 1.587, *p* > 0.22, $\eta_{\text{p}}^{2}$ < 0.045). We next analyzed food cup-directed behavior using the same factors. While there was no Sex main effect (*F*_(1,34)_ = 0.202, *p* = 0.656, $\eta_{\text{p}}^{2}$ = 0.006) for foodcup approach, we observed a Value × Tracking Group × Sex interaction, indicating that male and female ST rats show a different pattern of devaluation sensitivity than male and female GT rats (Figure 3G; *F*_(1,34)_ = 5.02, *p* = 0.032, $\eta_{\text{p}}^{2}$ = 0.114). For the foodcup approach, all other non-significant interactions with Sex had small effect sizes (Figure 3H; all *F* < 3.239, *p* > 0.081, $\eta_{\text{p}}^{2}$ < 0.068).

### Histological Verification

Figure 4 shows a summary of histological verification and representative examples of viral expression in the NAc core (Figures 4A,B) and BLA (Figures 4C,D) for hM4Di and mCherry constructs. Contralateral injections were counterbalanced, thus for each rat, only unilateral cell body expression was observed in contralateral BLA and NAc. The expression is shown in both hemispheres to represent both counterbalanced groups.

## Discussion

We examined the effect of contralaterally inactivating BLA and NAc core on devaluation sensitivity in sign- and goal-tracking rats. We found BLA–NAc core inactivation promoted devaluation sensitivity of sign-tracking rats and disrupted the devaluation sensitivity of goal-tracking rats. In viral control rats, we replicated previous findings that intact GT rats respond less to cues when the associated outcome is devalued, while ST rats respond at similarly high levels to cues regardless of outcome value (Keefer et al., 2020; Nasser et al., 2015). The tracking specificity of devaluation sensitivity has been observed across several studies, Pavlovian paradigms, and devaluation procedures (Nasser et al., 2015; Patitucci et al., 2016; Smedley and Smith, 2018; Keefer et al., 2020, but see Davey and Cleland, 1982; Derman et al., 2018; Amaya et al., 2020). In our study using both males and females, BLA–NAc core contralateral chemogenetic inactivation specifically reduced lever directed behavior, but not food cup-directed behavior, consistent with a prior BLA–NAc cross lesions study showing greater attenuation of lever directed approach in male rats (Chang et al., 2012). While we included both sexes, further studies would be needed to probe sex differences on the role of BLA–NAc communication in driving devaluation sensitivity.

A body of amygdala lesion and inactivation studies examining the neurobiology of incentive learning (for review see Wassum and Izquierdo, 2015) implicate candidate circuitry that may underlie differences in incentive learning that rely on the motivational properties of cues relative to the current value of the outcome. In brief, pre-training lesions of the BLA impair both the initial acquisition of incentive cue properties as well as subsequent updating of behavior in response to changing outcome values (Hatfield et al., 1996). Post-training lesions of the BLA similarly disrupt behavioral updating during devaluation (Johnson et al., 2009). Additionally, BLA lesions disrupt acquisition of positive incentive value (Setlow et al., 2002), while lesions of NAc prevent the expression of incentive value (McDannald et al., 2013) in SOC, and this pathway is necessary to acquire and express learned motivational value (Setlow et al., 2002). Disconnection of the BLA and NAc also produces deficits in both initial acquisition and expression of lever directed behavior, the preferred response of sign-tracking rats (Chang et al., 2012). Thus, we predicted that if ST rats rely on BLA to NAc communication to form rigid, behaviorally inflexible incentive value representations, then inactivation of BLA and NAc core would facilitate devaluation sensitivity. Consistent with our hypothesis, we observed that BLA–NAc inactivated ST rats showed better conditioned discrimination based on current outcome value than intact ST rats, which we interpret as more flexible behavior with chemogenetic inhibition. Because of the lower level of responding in BLA–NAc inactivated ST rats, it is more challenging to infer whether our chemogenetic manipulation promotes responding when appropriate, or suppresses responding when inappropriate. Regardless, our findings suggest that ST rats rely upon BLA and NAc to support rigid appetitive approach expressed as lever directed behavior.

Consistent with previous work, we observed that intact GT rats displayed devaluation sensitivity, reducing their preferred responding following outcome devaluation, while intact ST rats did not (Morrison et al., 2015; Nasser et al., 2015; Keefer et al., 2020). However, we found GT rats with BLA–NAc chemogenic inactivation were insensitive to devaluation. Notably, GT rats, on average showed similarly low levels of responding during the valued and devalued tests. This finding suggests that GT rats rely upon BLA and NAc to integrate and/or express information about the specific outcome they are sated on to promote responding when the outcome is valued. This loss in GT devaluation sensitivity seems to be an impairment in responding to cues when appropriate (i.e., valued condition) and not for suppressing responding when inappropriate (i.e., devalued condition). In a PLA task designed to promote goal-tracking responses, the NAc core is also necessary for the expression of goal-tracking (Blaiss and Janak, 2009). The present findings are also consistent with prior studies demonstrating that the BLA (Hatfield et al., 1996) and NAc (Singh et al., 2010) are critically involved in Pavlovian outcome devaluation. Additionally, the disconnection of the BLA and NAc produces a deficit in an instrumental outcome devaluation task (Shiflett and Balleine, 2010). The present study supports the role of this circuit in Pavlovian devaluation and suggests it may support different associative constructs in different individuals. That is, sign-trackers may rely on BLA and NAc to respond to cues based on their appetitive motivational properties, while goal-trackers rely on this circuitry to respond to cues based on the current value of the outcome. Consideration of tracking-specific behavioral and neurobiological differences, as in the present study, may provide a useful framework for interpreting individual variability in circuit manipulation studies.

The tracking-specific role of BLA and NAc core presented here falls into context with prior electrophysiological recording and optogenetic studies. Without BLA excitatory input, NAc fails to represent previously acquired CS-US associations, which blunts conditioned responding directed at both cues and outcomes (Ambroggi et al., 2008; Stuber et al., 2011). Compared to goal-trackers, sign-trackers show attenuated NAc reward signaling and stronger cue-evoked firing as training progresses (Gillis and Morrison, 2019). Similarly, NAc core cue-encoding during SOC positively correlates with SOC performance (Saddoris and Carelli, 2014). Surprisingly, ST and GT rats similarly acquire and express SOC (Saddoris and Carelli, 2014; Nasser et al., 2015), which seems somewhat at odds with the perspective that SOC and ST reflect similar positive incentive learning processes, both of which rely on BLA–NAc communication. Notably, enhanced NAc core cue encoding is also associated with better devaluation performance and sensory preconditioning, two learning processes that reflect an inference about either the current value of the outcome or value-independent predicative stimulus relationships (Cerri et al., 2014; West and Carelli, 2016). The pattern of results we observe in the present study, in which BLA–NAc core inactivation impedes devaluation sensitivity in ST rats, but facilitates devaluation sensitivity in GT rats, suggests individual or methodological differences that bias CS or US processing may account for the diverse role for BLA–NAc in incentive learning processes.

### Methodological Considerations

Our inclusion of both male and female rats is consistent with current best practices in neuroscience research and is part of a larger, growing trend to improve the representation of female subjects in basic science (McCarthy et al., 2017; Miller et al., 2017; Shansky, 2019). For practical reasons, we included both males and females without fully powering sex as a factor to test our hypothesis about the contribution of BLA and NAc in driving tracking-specific differences in devaluation sensitivity. Consistent with previous work, we observed that females displayed more lever directed behavior than males overall (Madayag et al., 2017; but see Pitchers et al., 2015; Bacharach et al., 2018). While the primary objective of this study was to include both sexes, not to probe sex differences, our analyses suggest that some sex effects may warrant further investigation. The present approach to include and report effects for both sexes ensures we do not rely solely on male rats to determine the causal role of brain circuit contributions to behavior.

The present work does not include the ipsilateral control group that is typical of traditional disconnection designs. In brief, our work employs contralateral chemogenetic inactivation of the BLA and NAc core. To demonstrate that effects are attributable to disrupted BLA–NAc core communication, rather than inactivation of these two regions alone, an ipsilateral control (in which communication between the structures is still possible unilaterally) is often employed. For practical reasons, we were unable to include an ipsilateral control group. However, we are not the first to contralaterally inactivate these regions, and a body of evidence demonstrates no effect of the ipsilateral disconnection of the BLA and NAc in similar tasks. Contralateral disconnection of the BLA and NAc disrupts the lever-directed approach in PLA both early and late in training. Critically, ipsilateral controls performed similarly to sham lesioned rats, suggesting unilateral functional communication between BLA and NAc is sufficient to support lever directed behavior (Chang et al., 2012). The present contralateral manipulations replicate the disconnection findings (Chang et al., 2012), bolstering our conjecture that BLA to NAc core communication is what drives our reported effects. Similarly, the ipsilateral disconnection of the BLA and NAc produces no impairment in instrumental outcome devaluation or Pavlovian instrumental transfer (Shiflett and Balleine, 2010). Additionally, anatomical evidence establishes BLA to NAc connectivity being primarily unidirectional and unilateral (Swanson and Cowan, 1975; Ottersen, 1980; Russchen and Price, 1984; Heimer et al., 1991; Brog et al., 1993; Kelley et al., 1993). Indeed, excitatory input (either direct or *via* modulation of dopaminergic inputs) into the NAc originating from the BLA drives neuronal responses to reward-predictive cues (e.g., Floresco et al., 2001; Ambroggi et al., 2008; Simmons and Neill, 2009; Jones et al., 2010). While disconnection of the BLA and NAc reduces neuronal excitability within the NAc and decreases responding toward reward-predictive cues, ipsilateral controls show significantly less pronounced (Ambroggi et al., 2008; muscimol/baclofen inactivation of BLA and D1 antagonism in NAc) or absent changes in excitability and reward-seeking behavior (Simmons and Neill, 2009; muscimol inactivation of BLA and D1/D2 antagonism in NAc).

The contralateral chemogenetic disconnection design does not preclude the possibility that a multisynaptic circuit could be involved in the reported effects. There could be a third contributing area, for example, the orbitofrontal cortex/insular cortex (OFC/IC), which receives projections from BLA and sends projections to NAc. The OFC/IC plays a causal role in PLA and Pavlovian outcome devaluation effects and is a likely candidate region at play if the presently reported effects are not due to direct communication between BLA and NAc (Pickens et al., 2003, 2005; Nasser et al., 2017). For feasibility reasons, we did not include both viral (mCherry, hM4Di) and ligand (clozapine, vehicle) control groups. We prioritized the viral control and gave all rats low-dose clozapine during devaluation and consumption tests to ensure any non-specific effect low dose clozapine would be detected in the viral control mCherry group. Importantly, low dose clozapine did not impair rats’ ability to discriminate sated from the non-sated outcome in choice consumption tests performed immediately after devaluation tests. Future studies using ligand control would provide an opportunity to detect tracking-related devaluation sensitivity differences in hM4Di expressing rats.

Altogether, while we expect the effects reported here reflect a disruption of communication from BLA to NAc, the ipsilateral control experiments would be necessary to confirm. We conclude that contralateral inactivation of BLA and NAc reveal opposite effects on devaluation sensitivity in sign- and goal-trackers.

### Conclusions

Pre-clinical studies evaluating behavioral and neurobiological markers of addiction-vulnerable individuals before any drug exposure are an important step toward understanding human addiction. Pre-clinical studies implicate BLA–NAc core communication in driving cocaine seeking (Di Ciano and Everitt, 2004), and NAc is heavily implicated in both sign-tracking and the enhanced cocaine relapse observed in ST rats (Flagel et al., 2011; Chang et al., 2012; Clark et al., 2013; Saunders et al., 2013; Fraser and Janak, 2017). Sign-trackers show an array of behaviors indicative of maladaptive incentive learning, including resistance to extinction (Ahrens et al., 2016; Fitzpatrick et al., 2019), heightened tolerance for negative consequences (Saunders and Robinson, 2010), and heightened attraction and sensitivity to the reinforcing properties of predictive cues (Flagel et al., 2007; Robinson and Flagel, 2009; Bacharach et al., 2018). While both ST and GT acquire the predictive relationship between cue and reward, ST is thought to attribute a higher level of incentive salience to the cue (Flagel et al., 2009; Pitchers et al., 2015; Flagel and Robinson, 2017). Sign-trackers’ inflexibility prior to and after drug experience (Saunders et al., 2013; Keefer et al., 2020) highlights the utility of the sign-tracking model for understanding the brain basis of SUD vulnerability. This work has translational relevance, as humans also show variability in cue reactivity and devaluation sensitivity (e.g., Garofalo and di Pellegrino, 2015; Versace et al., 2016; De Tommaso et al., 2017; Pool et al., 2019). A deeper understanding of the psychological and neurobiological differences present before drug exposure can enhance potential therapeutic interventions (e.g., Saunders and Robinson, 2010, 2013; McClory and Spear, 2014; Versaggi et al., 2016; Pitchers et al., 2017; Valyear et al., 2017). This work also underscores the importance of considering tracking- and sex-specific effects in neurobiological examinations of outcome devaluation. Future studies should be adequately powered to consider sex as a variable, as the present work suggests that there are important sex differences in flexibility that are relevant to addiction vulnerability.

## Data Availability Statement

The raw data supporting the conclusions of this article will be made available by the authors, without undue reservation.

## Ethics Statement

The animal study was reviewed and approved by University of Maryland, School of Medicine Institutional Animal Care and Use Committee.

## Author Contributions

DC conceived and supervised the project. DK, SK, and UG acquired the data. DK analyzed the data. DK and DC designed the experiments, interpreted the data, and wrote the manuscript. All authors contributed to the article and approved the submitted version.

## Conflict of Interest

The authors declare that the research was conducted in the absence of any commercial or financial relationships that could be construed as a potential conflict of interest.
